# Rapid, non-invasive, visual point-of-care detection of koi herpesvirus using loop-mediated isothermal amplification

**DOI:** 10.3389/fimmu.2026.1852824

**Published:** 2026-07-02

**Authors:** Na Jiang, Jianlin Luo, Chuiyu Kong, Zhihong Ma, Lin Luo, Xingchen Huo, Yan Chen, Huanhuan Yu, Jian Gao, Jinjing Zhang, Jinyou Hu, Dongjie Shi

**Affiliations:** 1 Fisheries Science Institute, Beijing Academy of Agriculture and Forestry Sciences, Beijing, China; 2 Key Laboratory of Urban Agriculture (North China), Beijing Academy of Agriculture and Forestry Sciences, Beijing, China; 3 College of Biological and Environmental Engineering, Guiyang University, Guiyang, China; 4 College of Engineering, China Agricultural University, Beijing, China; 5 College of Fisheries, Huazhong Agricultural University, Wuhan, China

**Keywords:** common carp and koi, koi herpesvirus, loop-mediated isothermal amplification (LAMP), non-invasive sampling, point-of-care test (POCT), visual testing

## Abstract

**Introduction:**

Koi herpesvirus (KHV or CyHV-3) causes a highly contagious and lethal disease in common carp and koi, resulting in substantial economic losses in global aquaculture. Rapid point-of-care testing (POCT) of KHV is critical for curbing its spread and preventing outbreaks. However, conventional diagnostic approaches, such as TaqMan quantitative PCR (qPCR), require professional personnel and specialized equipment, limiting their on-site applicability.

**Methods:**

A rapid visual POCT assay was developed by combing loop-mediated isothermal amplification (LAMP) with non-invasive mucus swab sampling. Two optimized release solutions enabled one-step nucleic acid preparation within 5 min. Among five primer sets designed, the optimal set targeting the thymidine kinase (TK) gene was selected for isothermal amplification at 65 °C for 60 min. The assay was further adapted for naked-eye result interpretation using a pH-sensitive indicatior dye.

**Results:**

The assay demonstrated a detection limit of 21.42 copies/μL, with a 95% confidence interval of 14.88—41.83 copies/μL. No cross-reactivity was observed with seven common fish pathogens. Comparative evaluation with TaqMan qPCR using parallel clinical specimens yielded 100% concordant results. The total detection time was 65 min, enabling the POCT implementation across diverse scenarios.

**Discussion:**

This non-invasive, visual LAMP-based POCT platform provides a sensitive, specific and equipment-free diagnostic strategy for KHV detection, facilitating timely disease surveillance, outbreak control management, and aquaculture biosecurity.

## 1 Introduction

The koi herpesvirus (KHV), also known as cyprinid herpesvirus 3 (CyHV-3), is recognized as one of the most virulent pathogens responsible for widespread disease outbreaks in common carp (*Cyprinus carpio* carpio) and koi (*Cyprinus carpio* koi). The disease caused by KHV, referred to as koi herpesvirus disease (KHVD), has been reported since 1999 by researchers from Europe, Israel, the USA, and East Asia (1–7), which are characterized by extremely high morbidity rates (80%-100%) and mortality rates (approximately 80%), resulting in substantial economic losses for the global aquaculture industry. The virus has been successfully isolated from organ homogenates through inoculation onto susceptible cell lines, including *Cyprinus carpio* brain (CCB), *Cyprinus carpio* gill (CCG) (3) and koi fin (KF-1) cells (4).

To date, a variety of diagnostic approaches have been developed for the detection of KHV, which can be categorized into two major types: immunological assays and molecular biological detection methods, both of which offer the advantages of speed and sensitivity. Since 2003, the enzyme-linked immunosorbent assay (ELISA) has been developed and evaluated for the detection of KHV-specific antibodies or antigens (8–12). Concurrently, several molecular biological detection techniques have been established, including conventional PCR (13), nested PCR (14, 15), hemi-nested PCR (16) and TaqMan qPCR (17). Although these assays demonstrate high specificity and sensitivity, their application necessitates trained personnel and specialized equipment. The novel technique of loop-mediated isothermal amplification (LAMP) (18) holds significant promise for on-site testing, offering rapid, sensitive, and specific amplification. Its primary advantage is that the entire reaction occurs at a constant temperature between 60 and 65 °C (19), eliminating the need for professional personnel or specialized equipment.

Several visualization-based methods have been developed for detecting LAMP amplicons, including turbidity assessment, fluorescence detection, lateral flow dipstick (LFD) assays, and colorimetric assays. However, the assessment of turbidity continues to pose a challenge due to a lack of sustained stability or clear discernibility for result interpretation. Fluorescent dyes, including SYBR Green I (20), calcein (21), and propidium iodide (22), provide high sensitivity but are expensive and require specialized ultraviolet (UV) equipment for their application, making them impractical for point-of-care testing (POCT). While LFD allows for the visual readout of LAMP results, they involve additional operational steps that may demand higher levels of professional expertise from testers. Colorimetric assays facilitate the interpretation of results achievable through direct visual observation. A variety of dyes have been developed for this purpose, including hydroxynaphthol blue (HNB) (23) and neutral red (NR) (24). HNB is not suitable for individuals with color blindness, because it exhibits only a subtle color changes, complicating result discrimination for these individuals. In contrast, NR demonstrates a distinct color transition from yellow to red, facilitating the visual discrimination of results.

In recent decades, LAMP has been applied for the detection of KHV and has demonstrated high specificity and sensitivity under controlled laboratory conditions (18, 20, 25). Nevertheless, a rapid POCT protocol that facilitates visual readout with the naked eye by untrained operators is still lacking. Such a platform would eliminate the need for specialized equipment and facilitate large-scale evaluation of vaccinated fish under farm conditions. Therefore, the present work aimed to establish and validate a KHV-POCT detection platform, providing a robust technical foundation for field-based assessment of fish vaccine efficacy and rapid detection of viral pathogens. Furthermore, this platform may support subsequent *in vivo* studies investigating fish immune responses. In this study, an innovative release solution and a visual LAMP reaction system were developed to establish a comprehensive POCT assay for the detection of KHV. Additionally, the performance of this newly developed method was assessed and compared with that of the TaqMan qPCR assay.

## 2 Materials and methods

### 2.1 Animal and fish welfare

The animal experiments conducted in this study were approved by the Animal Welfare and Ethics Committee of Beijing Academy of Agriculture and Forestry Sciences (Approval Code: BAAFS-KJLL-20250802). All animal experiments were performed in strict compliance with the “Guidelines for Experimental Animals” as stipulated by the Ministry of Science and Technology (Beijing, China). All samples (koi fish infected KHV or healthy fish) used in this study were obtained based on informed consent from farm owners. Control fish were sourced from farms free of KHV infection and showed no clinical symptoms. Additionally, TaqMan qPCR recommended by the World Organisation for Animal Health (WOAH) was performed on tissue samples to confirm the control fish were KHV-negative.

### 2.2 Sampling and DNA extraction from tissue and mucus specimens

After anesthesia with MS-222, samples of gill and intestinal tissues were collected from naturally infected and healthy koi. Gill mucus and anal mucus samples were collected from the same fish. The tissue samples were homogenized for LAMP and qPCR analysis. Meanwhile, mucus samples were lysed to release nucleic acids, which were subsequently utilized for the same two analysis. To collect gill and anal mucus samples for analysis, the gill and anal surfaces were initially rinsed with 500 μL of phosphate-buffered saline. Subsequently, a sterile swab was used to wipe and collect the mucus through a back-and-forth rotational motion. The swab was then incubated in one of the release solutions for 5 min at room temperature. Then the supernatant was aspirated and stored in Eppendorf tubes at -80 °C. The tissue samples were preserved in Eppendorf tubes at -80 °C.

Total DNA was extracted from 30 mg of tissue sample using the DNeasy blood and tissue kit (Qiagen, Hilden, Germany) following the manufacturer’s instructions.

### 2.3 Selection of KHV-release solution

The KHV-release solution is formulated to liberate nucleic acids from mucus samples, facilitating subsequent amplification via qPCR and LAMP assays. The compositions of these solutions are listed in **Table 1**. Both positive and negative samples were tested using these solutions. Additionally, the mucus samples, from which DNA was extracted and subsequently used for qPCR amplification, served as the standard for validating the KHV detection results.

**Table 1:** Components of KHV nucleic acids release solution.

Components	Final concentration		
	Release solution 1	Release solution 2	Release solution 3
Sodium hydroxide	2 mM	3 mM	3mM
Tween 20	0.1% (v/v)	0	0.1% (v/v)
Triton X-100	0	0.1% (v/v)	0
PEG 8000	0.05% (w/w)	0	0
NP-40	0	0.5% (w/w)	0
Tris-HCl buffer (pH 7.5)	0	5 mM	0
Tris-HCl buffer (pH 8.0)	10 mM	0	10 mM
Sodium dodecyl sulfate	0.5 mM	0	0.5 mM
Sodium citrate	20 mM	20 mM	20 mM
Ammonium sulfate	0	20 mM	0
Guanidine hydrochloride	2mM	2mM	2mM
EDTA solution	0.5 mM	0.5 mM	0.5 mM
dd H_2_0	up to the final volume of 20 mL	up to the final volume of 20 mL	up to the final volume of 20 mL

### 2.4 Design of LAMP primers and positive plasmid

Primers were designed based on the consensus sequence of TK genes in KHV (14 Asian and six European strains), retrieved from GenBank of the National Center for Biotechnology Information (NCBI). Sequence alignment was performed, and a consensus sequence was generated using BioEdit software (version 7.7.1).

The LAMP primers were manually designed following the guidelines established by Notomi (19). Each primer set comprised two outer primers (F3 and B3), two inner primers (FIP and BIP), and two loop primers (F-loop and B-loop). In total, five primer sets were designed (**Table 2**). All primers were synthesized by BGI Genomics (Beijing, China).

**Table 2:** Primer sets of LAMP assay.

Primer set name	Primer name	Sequence (5’-3’)
KHV-LAMP1	KHV-TK-F3-1	AACGCGGGCCAGCTG
	KHV-TK-B3-1	CCTTTTTCAGAGTGTGCGG
	KHV-TK-FIP-1	CGCGATTAAGTGGTTGGCATCttttCATTTTTGTGAAGTTGACAGGTTG
	KHV-TK-BIP-1	CGCAGCTCCTAGAGCCGCttttGGTGGTGCGGCTGCGA
	KHV-TK-Floop-1	GCCCTCTCAACACTTTGTCA
	KHV-TK-Bloop-1	GCTGCGGGCCCTACGC
KHV-LAMP2	KHV-TK-F3-2	GGGCCAGCTGAACATTTTT
	KHV-TK-B3-2	CATGGGTCCGATCACCAG
	KHV-TK-FIP-2	GCTCTAGGAGCTGCGCGAAAttttCAGGTTTGACAAAGTGTTGAGA
	KHV-TK-BIP-2	CTGCGGGCCCTACGCCttttCATAGCCATCCTTTTTCAGAG
	KHV-TK-Floop-2	GCGATTAAGTGGTTGGCATC
	KHV-TK-Bloop-2	TCGCAGCCGCACCACC
KHV-LAMP3	KHV-TK-F3-3	GGGCAAGAGCACAGAGAGC
	KHV-TK-B3-3	AACTGTCCCTCGTCGACG
	KHV-TK-FIP-3	GCATGGCCACCTTGGACTCTttttGGCTGGAGCGTCTGTCCTA
	KHV-TK-BIP-3	CACAGCGGCGCGACCTACttttCCTCCAGACGCTGCATCAC
	KHV-TK-Floop-3	CTATGGCGTGCTTGACGG
	KHV-TK-Bloop-3	CATCTCCGCGGGTTACCT
KHV-LAMP4	KHV-TK-F3-4	TCTTCCCCGACCTCTACGA
	KHV-TK-B3-4	TCGCGCATCTTGCACTT
	KHV-TK-FIP-4	GCTTGAAGGGCTGCTGCATttttCTGCTGACCGCGGGCAA
	KHV-TK-BIP-4	CGGCGTTGGTGCCCATttttGCACACCGCCGTCAGCT
	KHV-TK-Floop-4	AAAGTCCCCGTCCAGCG
	KHV-TK-Bloop-4	TGGCGGACAAGCTGGAC
KHV-LAMP5	KHV-TK-F3-5	CTGCTTGAAGGGCTGCTGC
	KHV-TK-B3-5	TATTGGACGCAAAGTCGCT
	KHV-TK-FIP-5	CGAGGTGGAGCGGCTGACAttttCCCAGTACGAGCTGTACGGT
	KHV-TK-BIP-5	TGTGATGGTGTGTGTGGAACCAAttttGCAAGTGTTTCGTGTTTCGG
	KHV-TK-Floop-5	CCAGTAGATTATGCGCAGGA
	KHV-TK-Bloop-5	TATTGGACGCAAAGTCGCT

A 997-bp fragment of the TK gene of KHV was synthesized and inserted into the pUC57 vector. This recombinant vector was then used to transform *Escherichia coli* (*E. coli*) DH5αcells, with the entire construction process conducted by BGI Genomics (Beijing, China). The resulting recombinant plasmid, designated as pUC57-TK, was used as the positive control plasmid for subsequent experimental assays. To accurately quantify this plasmid, its mass/volume concentration was measured, then converted it to molar concentration based on its theoretical molecular weight, and finally the copy number of plasmid per unit volume was calculated using Avogadro’s constant.

### 2.5 Optimization of amplification temperature for the LAMP assay

Each primer set was individually tested in the LAMP assay system at the concentration specified in **Table 3**. The total volume for the KHV-LAMP amplification system was 20 μL, including 10 μL of 2×LAMP Mix (20 mM KCl, 20 mM (NH4)_2_SO_4_, 16 mM MgSO_4_, 0.2% Triton X-100, 2.4 mM dNTPs, 40 mM Tris-HCl, pH 8.8), 1 μL of primer mix, 8 units of Bst DNA polymerase (New England Biolabs, Ipswich, USA), 1 μL of 20×Eva Green (UElandy, Suzhou, China), 1 μL of template, and nuclease-free water to achieve a final volume of 20 μL. The positive plasmid template was diluted to a concentration of 2 × 10^5^ copies/μL with TE buffer. The reaction system was incubated at 63 °C for elongation with 1 min per cycle, and fluorescent signals were collected after each cycle. Primer sets showing specific amplification were first identified based on the results. Then the optimal primer set for subsequent LAMP reactions was then selected by comparing the cycle threshold (Cycle to positivity, Cp) at which the positive signal first appeared. The whole amplification process comprises 60 cycles, and the results were analyzed using Roche LightCycler^®^ 480 II Real-Time qPCR System (Germany).

**Table 3:** Amount of LAMP primers in a 20-μL reaction system.

Primer name	Storage concentration (μM)	Volume per reaction (μL)	Concentration per reaction (μM)
F3	40	0.08	0.16
B3	40	0.08	0.16
FIP	100	0.32	1.6
BIP	100	0.32	1.6
F-loop	80	0.1	0.4
B-loop	80	0.1	0.4

The amplification temperature was optimized by conducting the reaction system at temperature of 60, 61, 62, 63, 64, 65, and 66 °C. This optimization was performed using the previously described LAMP reaction conditions and an optimized primer set. The experiments were repeated three times.

### 2.6 Analytical sensitivity and specificity of the LAMP assay

After optimizing the primers and amplification temperature, the analytical sensitivity of the LAMP assay was evaluated using 10-fold serial dilution of a positive plasmid (2 × 10^6^, 2 × 10^5^, 2 × 10^4^, 2 × 10^3^, 2 × 10^2^, 2 × 10^1^, and 2 × 10^0^ copies/μL), and nuclease-free water was used as negative control, with triplicates at each dilution.

To determine the 95% limit of detection (LOD_95%_), a 2-fold serial dilution of the positive plasmid (80, 40, 20, 10, 5, 2.5 copies/μL) was tested by the LAMP assay, with 16 replicates at each dilution. The LOD_95%_ values were calculated using MedCalc statistical software ver. 23.5.2. The probability of detection (POD) curve was generated with 95% confidence interval (CI).

To determine the analytical specificity of the LAMP assay, nucleic acids were extracted from seven types viral and bacterial pathogens affecting fish, sourced from either diseased fish tissues or fish cell lines. The fish cell lines employed were channel catfish ovary (CCO) and epithelioma papulosum cyprinid (EPC). The viral pathogens included channel catfish virus (CCV), carp edema virus (CEV), cyprinid herpesvirus 2 (CyHV-2 (26)), soft-shelled turtle iridovirus (STIV), red sea bream iridovirus (RSIV), and white spot syndrome virus (WSSV). The bacterial pathogen examined was *Aeromonas veronii* (*A. veronii*) (7231 (27)). The concentration of others pathogens’ DNA was between 45.68−470.52 ng/μL.

The experiments were repeated three times.

### 2.7 Analysis of the tissue samples using the LAMP and qPCR assays

To evaluate the efficacy of the LAMP assay, a total of 30 tissue samples were assessed for positivity through the qPCR assay. Genomic DNA was extracted from these samples, adhering to the protocols outlined for either the LAMP or qPCR assays. The LAMP assay was conducted as described in section 4.5. For the qPCR analysis, reactions were carried out according to previous established methods (19). Briefly, each TaqMan reaction mixture (25 μL total volume) comprised 400 nM of each primer, 80 nM of the TaqMan probe and 5 μL of the diluted DNA template, prepared using a commercial PCR Mastermix (TaqMan Universal PCR Mastermix, Applied Biosystems, Carlsbad, USA). The amplification conditions were set at 2 min at 50 °C, 10 min at 95 °C, 40 cycles of 15 s at 95 °C, and 60 s at 60 °C. Fluorescence signals were detected using Roche LightCycler^®^ 480 II Real-Time qPCR System (Germany).

### 2.8 Development of a visualized LAMP assay

The total volume of the visualized KHV-LAMP system was set of 20 μL, comprising 17 μL of MyLab^®^ visualized LAMP DNA detection kit (MyLab, Beijing, China), 1 μL of *Bst* DNA polymerase (8 Units), 1 μL of primer mix, and 1 μL of template. The primer mix concentration used in this reaction system was the same as that in section 4.5, and the reaction system was incubated at an optimized amplification temperature for 60 min. The results were assessed visually, with a red color indicating a positive result and a yellow color indicating a negative result.

Two independent sensitivity evaluations were performed for the visualized LAMP assay. First, a 10-fold serial dilution of the positive plasmid (2 × 10^6^, 2 × 10^5^, 2 × 10^4^, 2 × 10^3^, 2 × 10^2^, 2 × 10^1^, and 2 × 10^0^ copies/μL) was prepared in TE buffer and employed as templates. Nuclease-free water was used as negative control.

Second, an additional sensitivity evaluation was conducted using a spike-in sample model, where the positive plasmid control and negative tissue DNA (50 ng/μL) were mixed at a 1:1 ratio as templates. Specifically, 10-fold serial dilutions of the positive plasmid (4 × 10^6^, 4 × 10^5^, 4 × 10^4^, 4 × 10^3^, 4 × 10^2^, 4 × 10^1^, and 4 × 10^0^ copies/μL) were mixed with an equal volume of negative tissue DNA, and the resulting mixtures were employed as templates for visualized LAMP assay. The negative control consisted of a mixture of nuclease-free water and negative tissue DNA (50 ng/μL) at the same 1:1 volume ratio. All dilutions and controls in both evaluations were tested in triplicate to ensure reliability.

The specificity of the visualized LAMP assay was assessed using the same seven types of fish pathogens as those described in section 2.6.

All the assay experiments were repeated three times.

## 3 Results

### 3.1 Optimization of LAMP amplification conditions

**Figure 1:**
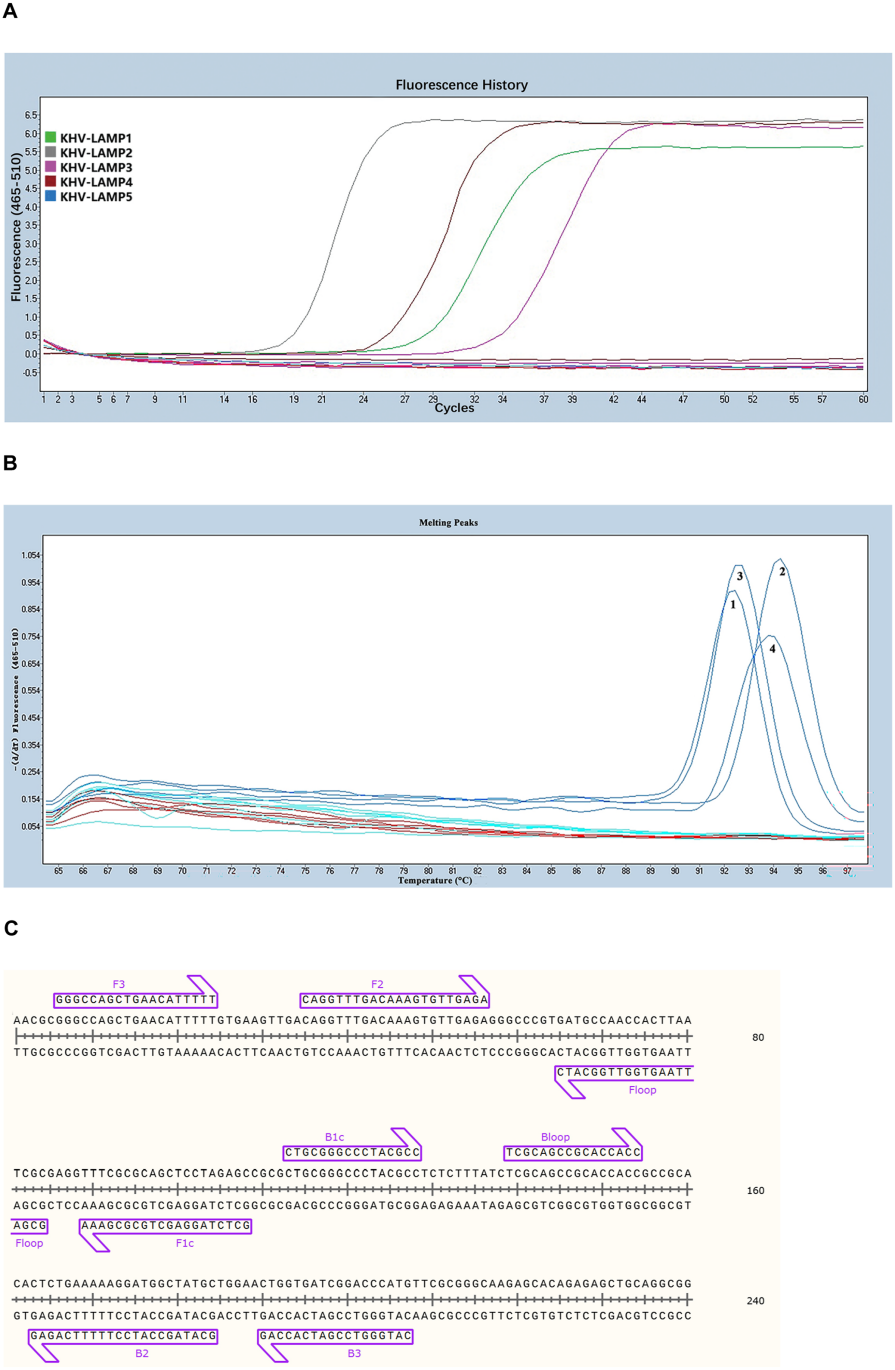
Experiment results showing the primer set with optimal amplification efficiency in LAMP assay and its genomic localization. **(A)** LAMP amplification curves of positive and negative controls using five sets of primers; **(B)** LAMP melting curves of the positive and negative controls using five sets of primers. The melting curves of the positive amplicons (from primer sets 1, 2,3, and 4) are labeled with Arabic numerals, while no labels are shown for the negative controls and primer set 5 in the figure due to the absence of specific amplification. The melting temperatures of the four positive amplification products are 92.5 °C, 94.2 °C, 92.7 °C and 93.9 °C, respectively; **(C)** Genomic location and sequence of the KHV-LAMP2 target region and primer-binding sites. Purple arrows indicate the DNA sequences used for LAMP primer design.

Five sets of primers (**Table 2**) were employed for the LAMP amplification of the positive plasmid using the methods described in section 2.5. The primer sets KHV-LAMP1, KHV-LAMP2, KHV-LAMP3, and KHV-LAMP4 successfully amplified the positive plasmid without cross-reactivity with the negative control (**Table 4**; **Figure 1**). In contrast, KHV-LAMP5 failed to amplify the positive control. Among the effective primer sets, KHV-LAMP2 demonstrated the highest fluorescence intensity and the lowest Cp value, indicating superior amplification efficiency (**Figure 1A**). Furthermore, the melting curve for KHV-LAMP2 displayed a single peak (**Figure 1B**), confirming the production of a specific and homogeneous amplification product.

**Table 4:** Amplification Cp value of five primer sets.

Primer name	Control name	Cycle to positivity (Cp)			
		Cp1	Cp2	Cp3	Mean of Cp
KHV-LAMP1	positive	28.23	29.28	26.98	28.16
	negative	nd *	nd	nd	nd
KHV-LAMP2	positive	19.6	19.06	19.46	19.37
	negative	nd	nd	nd	nd
KHV-LAMP3	positive	37.73	34.56	33.66	35.32
	negative	nd	nd	nd	nd
KHV-LAMP4	positive	26.9	26.32	25.16	26.13
	negative	nd	nd	nd	nd
KHV-LAMP5	positive	nd	nd	nd	nd
	negative	nd	nd	nd	nd

*nd means not detected.

The target gene for the LAMP assay was located within the thymidine kinase (*TK*) gene of KHV, whereas the target gene for TaqMan qPCR corresponded to the fragment associated with GenBank accession no. AF411803. The *TK* gene sequences of the 20 reference strains used for LAMP primer design were subjected to sequence alignment analysis (**Supplementary Figure 1**). The position of the selected primer set is illustrated in **Figure 1C**.

The amplification temperature was optimized as described in section 2.5. Among the seven tested temperatures, 65 °C yielded the lowest Cp value (**Table 5**).

**Table 5:** Amplification Cp value of different amplification temperature.

Amplification temperature (°C)	Control name	Cycle to positivity (Cp)			
		Cp 1	Cp 2	Cp 3	Mean of Cp
60	positive	18.45	20.43	19.43	19.44
	negative	nd *	nd	nd	nd
61	positive	16.57	15.80	16.42	16.26
	negative	nd	nd	nd	nd
62	positive	16.31	15.56	13.75	15.21
	negative	nd	nd	nd	nd
63	positive	15.61	15.30	15.16	15.36
	negative	nd	nd	nd	nd
64	positive	14.88	14.10	14.75	14.58
	negative	nd	nd	nd	nd
65	positive	14.39	14.18	14.64	14.40
	negative	nd	nd	nd	nd
66	positive	15.44	14.90	15.59	15.31
	negative	nd	nd	nd	nd

*nd means not detected.

### 3.2 Sensitivity and specificity of the LAMP assay

**Figure 2:**
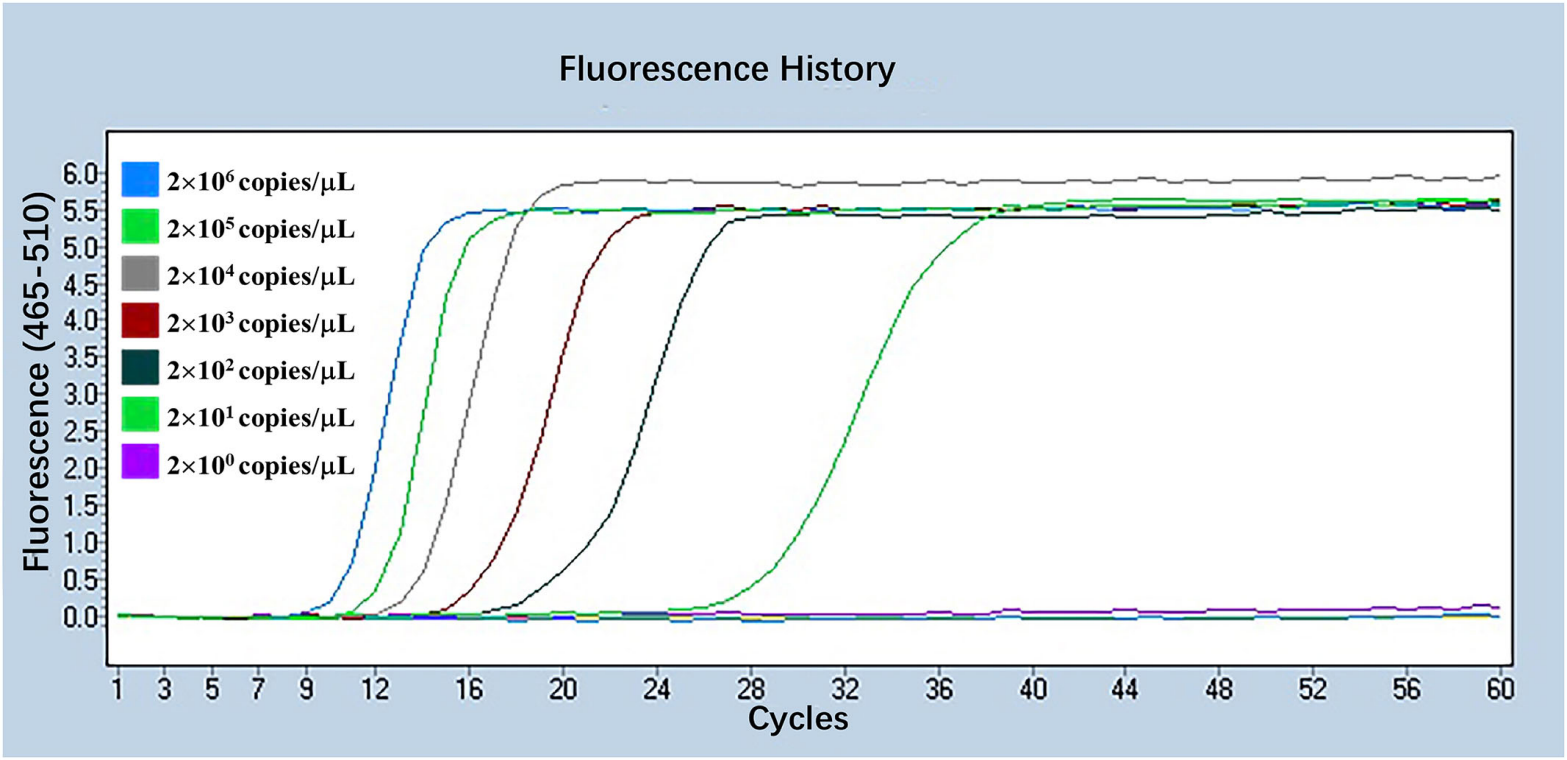
Amplification curves of the positive and negative controls with 10-fold serial dilutions of the KHV positive plasmid using the LAMP assay.

**Figure 3:**
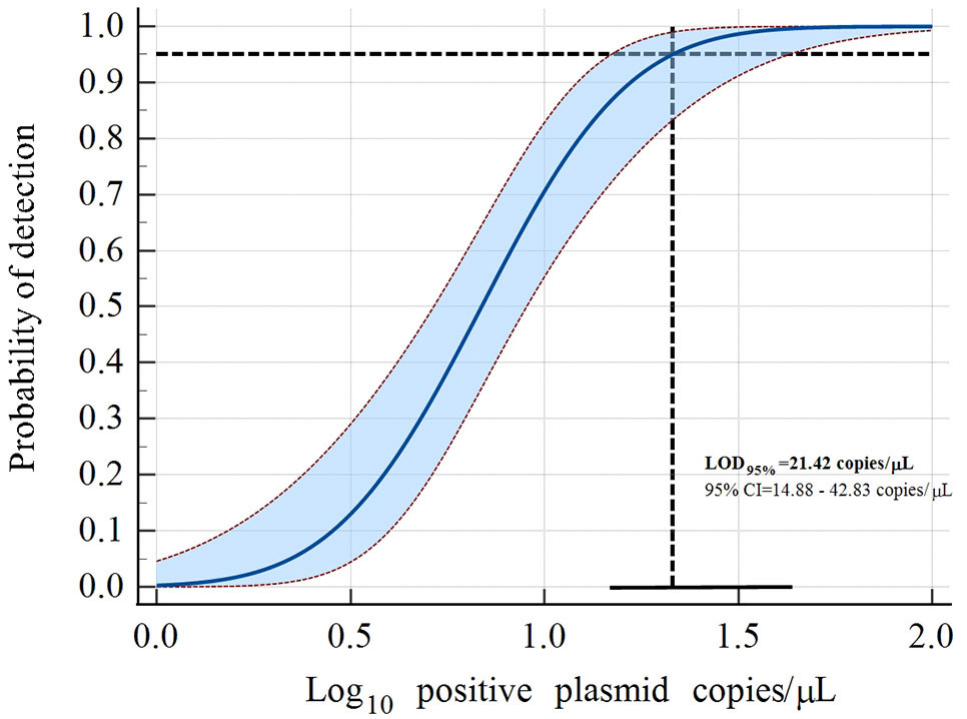
Probability of detection (POD) curve for LAMP assay. Solid line represents the mean POD, with the corresponding 95% confidence interval shaded in blue.

The sensitivity of the LAMP assay was firstly determined using 10-fold serial dilutions of the KHV-positive plasmid suspension. Utilizing a real-time fluorescence detector to monitor the LAMP reaction, the detection threshold for the positive plasmid, employing the KHV-LAMP2 primer set, was found to be 2 × 10^1^ copies/μL within a 60 min timeframe (**Table 6**; **Figure 2**). The LOD_95%_ of LAMP assay was 21.42 copies/μL, with a 95% CI of 14.88 to 42.83 copies/μL (**Figure 3**), as determined by Probit regression analysis following the method described in section 2.6.

**Table 6:** Amplification Cp value of serially diluted positive plasmid.

Concentration of positive plasmid (copies/μL)	Cycle to positivity (Cp)			
	Cp 1	Cp 2	Cp 3	Mean of Cp
2 × 10^6^	10.8	10.81	10.69	10.77
2 × 10^5^	12.63	12.49	12.79	12.64
2 × 10^4^	14.11	14.24	14.59	14.31
2 × 10^3^	16.71	16.5	16.92	16.71
2 × 10^2^	20.5	20.97	21.22	20.90
2 × 10^1^	28.23	29.28	31.21	29.57
2 × 10^0^	nd *	nd	nd	nd
dd H_2_0	nd	nd	nd	nd

*nd means not detected.

The specificity of the LAMP assay was examined using nucleic acids from seven distinct fish pathogens. Significant amplification was observed for both the KHV-positive plasmid and genomic DNA extracted from KHV-positive tissue when the LAMP-KHV2 primer set was employed. In contrast, no amplification was detected for the nucleic acids of the other seven fish pathogens after a 60-min incubation period (**Table 7**).

**Table 7:** Amplification Cp value of nucleic acids from different fish pathogens.

Name of fish pathogens	Cycle to positivity (Cp)			
	Cp 1	Cp 2	Cp 3	Mean of Cp
DNA of KHV-positive tissue	19.2	19.86	18.71	19.26
KHV-positive plasmid	21.53	19.94	20.62	20.70
CCV	nd *	nd	nd	nd
CEV	nd	nd	nd	nd
CyHV-2	nd	nd	nd	nd
STIV	nd	nd	nd	nd
RSIV	nd	nd	nd	nd
WSSV	nd	nd	nd	nd
*A.veronii*	nd	nd	nd	nd
dd H_2_0	nd	nd	nd	nd

*nd means not detected.

### 3.3 Comparative analysis of detection efficacy between LAMP and qPCR in tissue specimens

DNA was extracted from 30 tissue specimens (including gill and intestine tissues) using the method described in section 2.2, and the extracts were subjected to detection and analysis using both LAMP and qPCR methods (**Table 8**). All 24 specimens identified as positive by TaqMan qPCR were also detected as positive using the KHV-LAMP2 assay.

**Table 8:** Detection performance of tissue specimens via two assays.

Specimen name	Specimen type	LAMP (KHV-LAMP2)		TaqMan qPCR	
		Cp	Result	Ct	Result
1	gill	20.39	+	28.75	+
2	gill	19.19	+	29.95	+
3	gill	14.89	+	19.84	+
4	gill	15.93	+	22.32	+
5	gill	17.84	+	24.55	+
6	gill	18.57	+	26.35	+
7	gill	20.57	+	27.3	+
8	gill	13.83	+	21.58	+
9	gill	36.58	+	33.7	+
10	gill	31.02	+	26.05	+
11	gill	18.06	+	22.27	+
12	gill	nd *	–	nd	–
13	gill	17.21	+	20.86	+
14	gill	18.7	+	25.86	+
15	gill	16.78	+	21.5	+
16	intestine	nd	–	nd	–
17	intestine	17.18	+	20.68	+
18	intestine	16.26	+	21.47	+
19	intestine	nd	–	nd	–
20	intestine	21.5	+	25.31	+
21	intestine	18.66	+	26.95	+
22	intestine	19.89	+	28.4	+
23	intestine	nd	–	nd	–
24	intestine	16.78	+	24.05	+
25	intestine	16.22	+	21.74	+
26	intestine	31.64	+	32.08	+
27	intestine	nd	–	nd	–
28	intestine	15.57	+	24.07	+
29	intestine	19.85	+	28.59	+
30	intestine	nd	–	nd	–
Positive control	/	19.5	+	19.31	+
Negative control	/	nd	–	nd	–

*nd means not detected.

### 3.4 Development of the visualized LAMP assay

**Figure 4:**
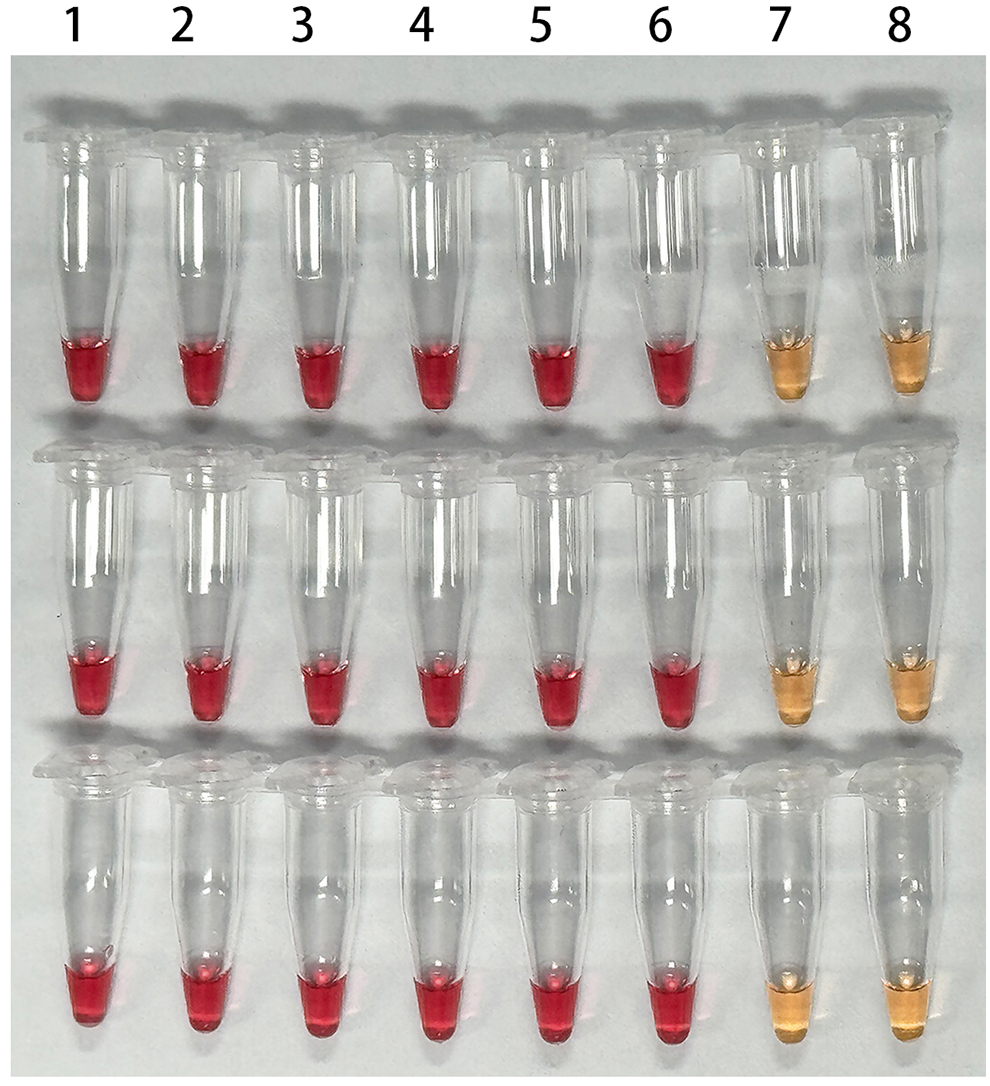
Detection of the KHV-positive plasmid (10-fold serial dilutions) and negative control using the LAMP assay. Lanes 1–6: KHV-positive plasmid at concentrations ranging from 2 × 10^6^ to 2 × 10^1^ copies/μL, all exhibiting a red color indicative of the presence of LAMP products in these reaction tubes; Lanes 7–8: KHV-positive plasmid at 2 × 10^0^ copies/μL and the negative control, showing a yellow color indicative of the absence of LAMP products in these reaction tubes.

The visualized LAMP assay was incubated at 65 °C and the optimization results are provided in section 3.1. The visualized LAMP assay demonstrated the capability to detect KHV-positive plasmid at a concentration of 2 × 10^1^ copies/μL (**Supplementary Table 1**; **Figure 4**).

**Figure 5:**
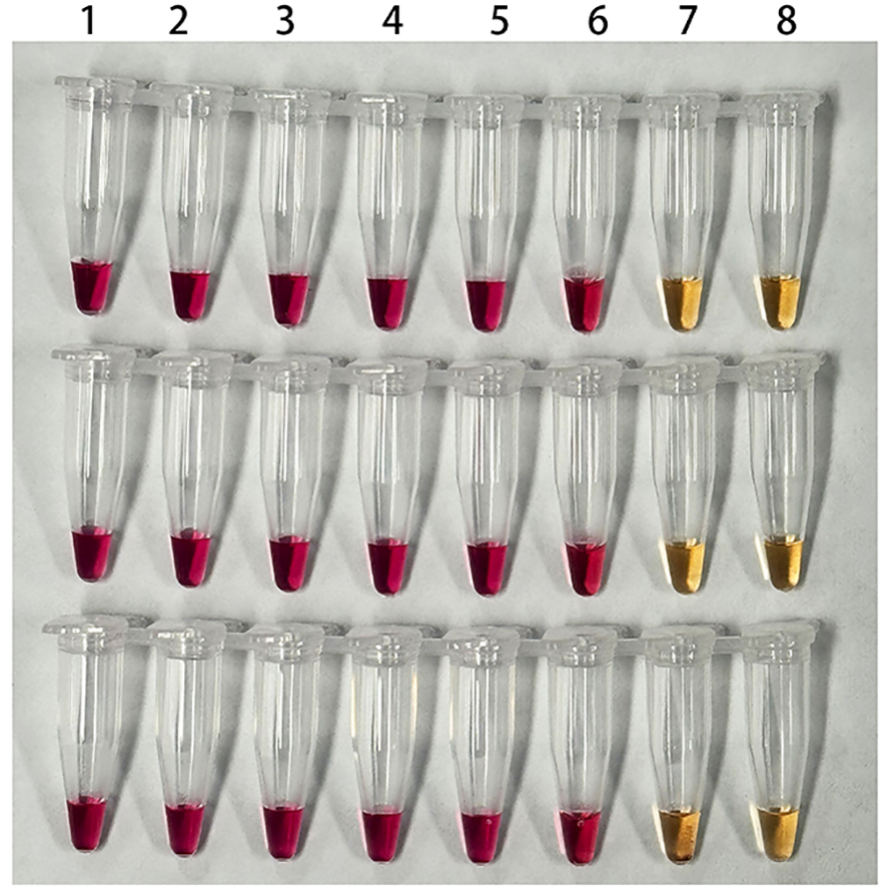
Detection of the KHV-positive plasmids (10-fold serial dilutions) in a spiked sample model using the LAMP assay. Lanes 1–6: KHV-positive plasmids at concentrations ranging from 2 × 10^6^ to 2 × 10^1^ copies/μL, all exhibiting a red color indicative of the presence of LAMP products in these reaction tubes; Lanes 7–8: KHV-positive plasmid at 2 × 10^0^ copies/μL and the negative control, showing a yellow color indicative of the absence of LAMP products in these reaction tubes.

In the spike-in sample model, the minimum concentration of the positive plasmids in the artificially simulated sample was 2 × 10^1^ copies/μL, and this result is consistent with the results previously obtained using standard positive plasmid dilutions (**Figure 5**).

**Figure 6:**
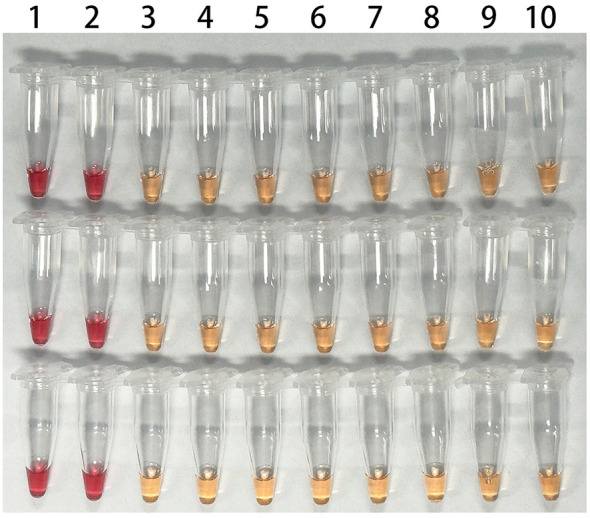
Specificity of the LAMP assay demonstrated by the visual detection of nucleic acids from different fish pathogens. Lanes 1–2: DNA from KHV-positive tissue and KHV-positive plasmid, showing a red color indicative of the presence of LAMP products in these reaction tubes; Lanes 3–10: DNA from CCV, CEV, CyHV-2, STIV, RSIV, WSSV, *A.veronii*, and negative control, showing a yellow color indicative of the absence of LAMP products in.

The specificity of the visualized LAMP assay was assessed using nucleic acids from seven different fish pathogens, consistent with the strains used for the specificity validation of the LAMP assay. Positive discoloration results were observed for both the KHV-positive plasmid and genomic DNA extracted from KHV-positive tissue samples. In contrast, coloration consistent of a negative result was observed for the nucleic acids of the other seven fish pathogens (**Supplementary Table 2**; **Figure 6**).

### 3.5 Optimization of KHV-release solution and its application in visualized LAMP assay

**Figure 7:**
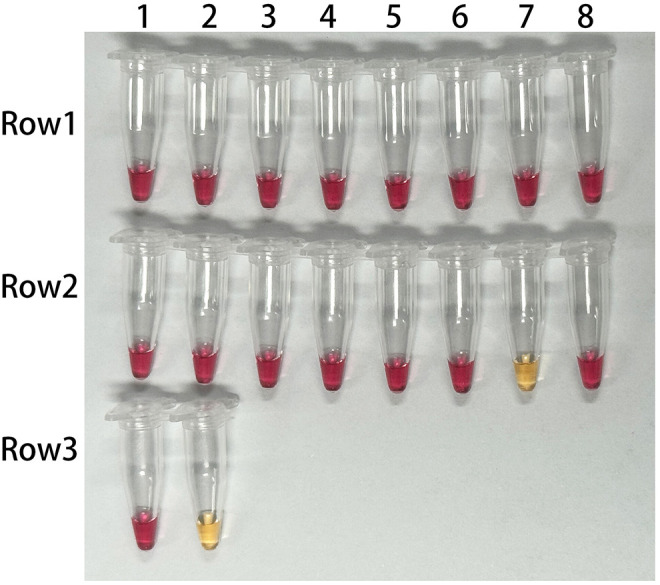
Visual detection of KHV in mucus specimens using four different DNA extraction methods. Row 1 and Row 2: present LAMP results of gill and anal swab samples 1 and 2. In Row 1, Lanes 1–4 correspond to gill swab specimen 1 and Lanes 5–8 to anal swab specimen1, with DNA extracted using Release solution 1, 2, 3, and a conventional kit in sequence. Row 2 shows the parallel results of specimen 2 using the same extraction methods. Row 3 includes the positive plasmid control (Lane 1); and the negative control (Lane 2).

DNA was extracted from all mucus samples using three distinct one-step release solutions (**Table 1**), with a conventional DNA extraction kit serving as the positive control. All tested solutions demonstrated the ability to release DNA, including viral DNA. However, the DNA sample extracted from one specimen using Release Solution 3 yielded a negative result in the TaqMan qPCR assay. Importantly, all mucus samples consistently showed amplification results with both conventional LAMP and visualized LAMP assays that were comparable to the outcomes obtained via TaqMan qPCR (**Supplementary Table 3**; **Figure 7**).

### 3.6 Determination of clinical specimens using visualized LAMP and qPCR assays

**Figure 8:**
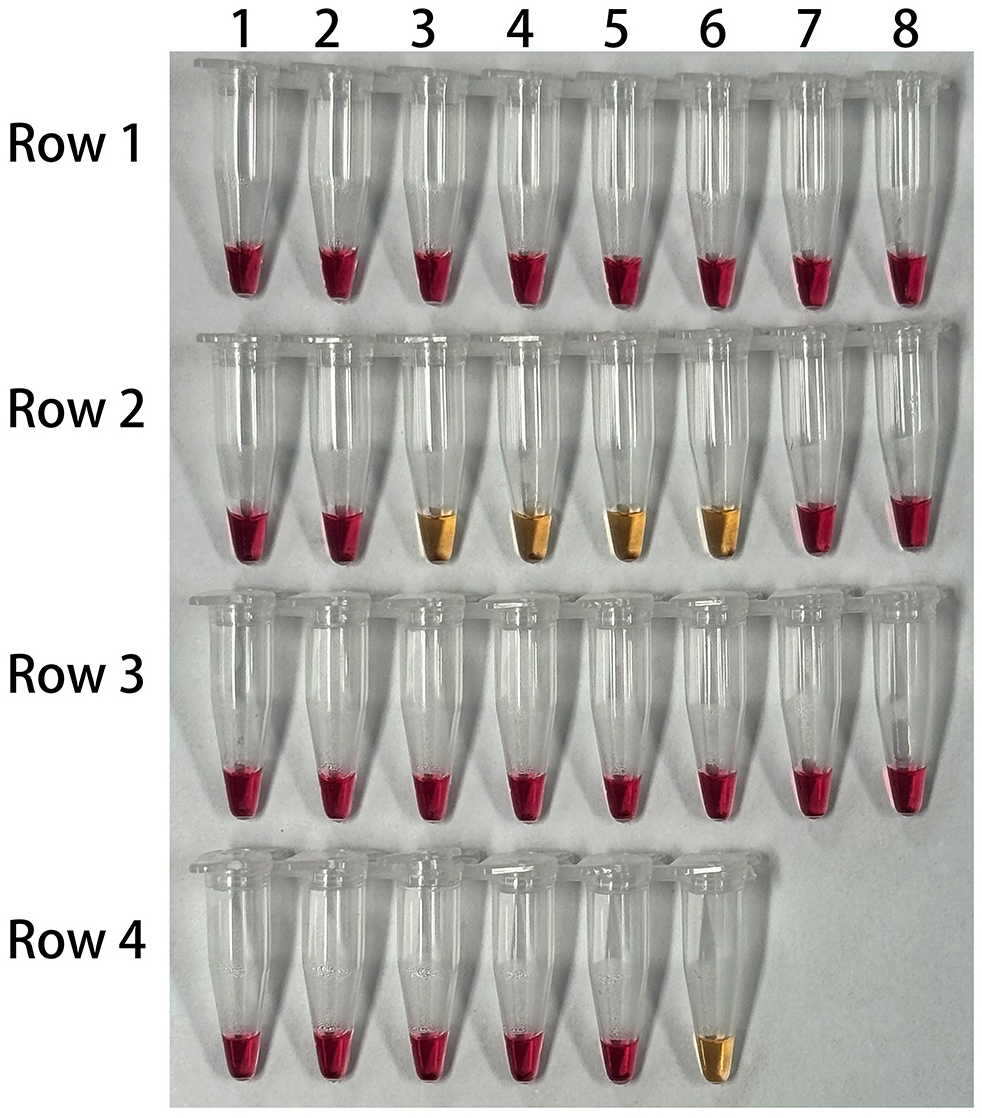
Visual LAMP assay of KHV in clinical specimens. Row 1–4 (except Lanes 5–6 of Row 4) show the LAMP results of DNA extracted from clinical specimens using a conventional extraction kit. Row 1 (Lanes 1–8) and Row 2 (Lanes 1–2) represent gill tissue samples from 10 fish; Row 2 (Lanes 3–8) and Row3 (Lanes 1–4) show the parallel detection results of intestine tissue specimens from the same fish individuals. Row 3 (Lanes 5–8) corresponds to gill mucus samples, and Row 4 (Lanes 1–4) shows the parallel results of anal mucus specimens. In Row 4, Lane 5 serves as the positive plasmid control (red color), and Lane 6 is the negative control (yellow color).

The visualized LAMP assay successfully detected the KHV DNA in 24 out of 28 clinical specimens tested, with the results aligning with those obtained using the TaqMan qPCR assay (**Supplementary Table 4**; **Figure 8**). These clinical specimens included DNA extracted from both tissue and mucus samples, and the tissue-derived DNA was isolated using a conventional DNA extraction kit, while mucus-derived DNA was prepared using Release Solution 1.

## 4 Discussion

**Figure 9:**
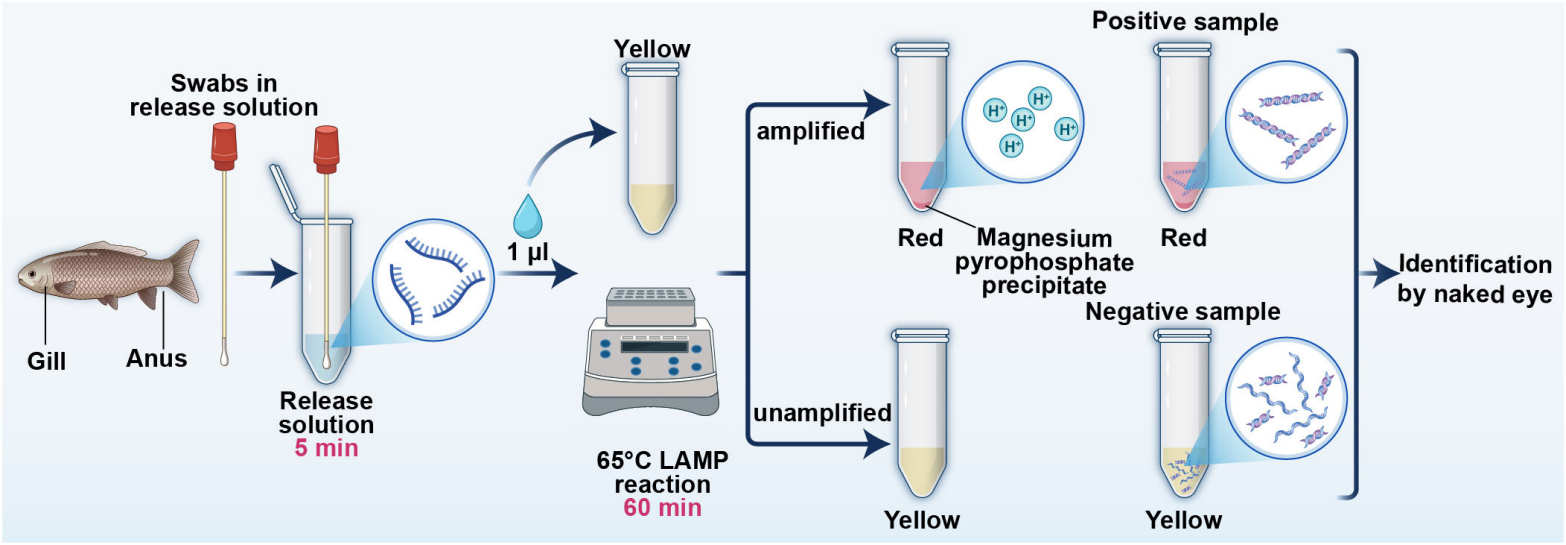
Schematic representation of the assay procedure. Gill and anal mucus samples were collected using swabs, which were then immersed in release solution for 5 min. Subsequently, 1 μL of the resulting lysate was transferred into an amplification tube and incubated at 65 °C for 60 min. The assay results were determined by changes in color.

The rapid and accurate on-site detection of viral infections in common carp and koi is essential for the effective management and control of KHV outbreaks in fish farms, as well as live-fish transportation, and ornamental koi family breeding. Previous studies have focused on developing innovative molecular biotechnology-based detection techniques and materials, including triplex PCR (28), cross-priming amplification-based lateral flow assay (CPA-LFA) (29), RAA-SHERLOCK assay (30), and LAMP combined with gold probes (AuNPs) and SYBR safe fluorescent dye (25). In contrast, in the present study, we emphasized enhancing the accuracy and visualization capabilities of the LAMP assay, as well as optimizing the convenience and efficacy of the sample processing protocols (**Figure 9**). The details of this protocol were described in **Supplementary Materials**. Notably, earlier research employed tissue sampling followed by nucleic acid extraction using conventional kits. However, this can cause injury and often mortality in the sampled fish. The present study addressed this limitation by employing non-invasive gill or anal mucus sampling, coupled with nucleic acid release via optimized lysis solutions. This approach facilitates the practical implementation of the KHV-POCT across diverse operational scenarios.

Since its inception, the LAMP assay has been characterized by its high sensitivity and specificity, despite the amplification and detection reactions proceeding in a single tube under isothermal conditions. In this study, the amplification efficiency was maximized when the reaction was continuously incubated at 65 °C. This temperature is consistent with that those applied in previous KHV detection studies (18, 20), as well as assays targeting Beiji nairovirus (31) and Alongshan virus (32). By contrast, the optimal temperature has been reported to vary across different pathogens. For example, 63 °C was found to be suitable for the detection of *Sclerotinia sclerotiorum* (33).

In this study, by investigating the sensitivity of conventional LAMP and visualized LAMP assays for KHV detection, both methods were found to exhibit an identical detection limit of 2 × 10^1^ copies/test. This value is substantially higher than those reported for conventional PCR (13, 34) and nested PCR (15), slightly higher than the detection limit of highly sensitive PCR for KHV reported previously (35), yet marginally lower than the detection limit of six copies/test documented for a prior LAMP-based detection system (18). The detection limit of the visualized LAMP assay in the present study was notably sensitive enough to identify KHV during the acute phase. As reported by Gilad (17), 58 out of 61 samples collected from fish mucus, gill, and intestinal tissues specimens during the acute phase contained more than 8.05 × 10^1^ KHV genome equivalents, whereas the remaining three samples were undetectable even with TaqMan qPCR. More specifically, the LOD_95%_ of the LAMP assay was determined using an experimental protocol with 16 replicates, substantially more replicates than those employed in previous studies on KHV amplification assays. This provide a solid foundation for improving the reliability and rigor of the detection performance. Moreover, the LOD_95%_ of the LAMP (21.42 copies/μL) was slightly higher than that of TaqMan qPCR (8.384 copies/μL), yet markedly lower than that of the cross-priming amplification-based lateral flow assay (CPA-LFA, 675.69 copies/μL).

In this study, both conventional and visualized LAMP assays demonstrated high specificity. This finding is consistent with specificity results reported for LAMP-SYBR safe and LAMP-AuNPs assays (25). To evaluate specificity, a panel of related and co-occurring pathogens was tested, including fish herpesviruses (e.g., CCV and CyHV-2), carp DNA viruses (e.g., CEV), and *A. veronii*, a prevalent bacterium that often causes secondary infections in KHV-infected fish. None of the DNA templates derived from these pathogens exhibited cross-reactivity with the LAMP systems, thereby confirming the high specificity of our LAMP assays for KHV detection.

Two high-efficacy nucleic acid release solutions were also optimized, which can be integrated with a visualized LAMP assay to enable POCT detection for KHV for non-invasive sampling. Compared to a third candidate release solution, these two effective formulations exhibited two key distinguishing features: the inclusion of either polyethylene glycol (PEG) or Nonidet P-40 (NP-40), and a relatively lower overall pH. Notably, PEG and NP-40 have been well-documented as enhancers for improving the amplification efficiency of high-GC templates (36). In our POCT strategy, the presence of either of these two components was essential for the effective functioning of the release solution. Regarding the second feature, the higher pH of Release solution 3, driven by the Tris-HCl buffer (pH8.0) and increased amounts of sodium hydroxide, may affect the visualized LAMP assay, as the initial pH is too high for phenol red color change (37). Additionally, the inclusion of Tween 20 or Triton X-100 was necessary for the PCR reaction, consistent with the release solution used for in *E. coli* DNA extraction from milk in a previous study (38). These two surfactants were presumed to have no inhibitory effects on PCR reactions, thereby potentially enhancing the activity of Taq DNA polymerase. Moreover, the optimized release solutions developed in this study demonstrated significant potential for integration with CRISPR-Cas12a system-based aquaculture virus platforms. This integration could provide substantial technical support for the development of novel POCT diagnostic assays.

This KHV-POCT assay integrates equipment-free and one-step operation. DNA extraction through spcimens requires no centrifuge machine, and amplification is performed without thermal cyclers or real-time PCR systems. Simple thermal incubation, even one thermometer plus one insulated flask suffices for the reaction. Furthermore, pH-sensitive dye enables direct readout of detection results, in contrast to SYBR Green dye, which is added post-amplification. This protocol avoids false positives caused by aerosol contamination of amplified positive products. Collectively, these features ensure a contamination-free operating environment and reliable detection outcomes. Although the cost of the LAMP assay is higher than that of conventional PCR and SYBR Green qPCR, its advantages, including rapid amplification, visual readout, minimal equipment requirements, and suitability for on-site detection, can offset the additional cost and enhance its practicality for field applications.

Nevertheless, several limitations should be acknowledged. First, all LAMP-based detection methods rely on one set of six primers, which incurs a higher cost than conventional PCR and SYBR Green quantitative PCR, both of which only require two primers each. Furthermore, these six primers must be purified by HPLC, which further increases the total cost of the primer set to approximately $0.35 per reaction. Second, neutral red may exhibit lower sensitivity than intercalating dyes (e.g., SYBR Green I), and the yellow-to-red color change introduces subjectivity into the judgment of positive results, thereby making it challenging to achieve reliable identification of low viral loads in practical validation. For clinical application, a two-round LAMP strategy may be adopted. Samples yielding uncertain results can be considered suspicious and further confirmed using complementary assays, such as TaqMan qPCR or ELISA. Third, further optimization of the primer set could be conducted across a gradient of reaction temperatures to expand the screening scope. This temperature-gradient screening strategy has the potential to further enhance amplification efficiency or even shorten the overall amplification time, thereby refining the assay performance. Additionally, to improve the practicality and convenience of the POCT platform for on-site application, the integration of a compact temperature-control module is deemed necessary in subsequent development. In total, 62 clinical specimens were included in this study. Although these samples provided initial support for evaluating the performance of the assay, we recognize that larger-scale clinical validation would further improve confidence in its robustness and reliability. Future studies involving a broader sample set will therefore be conducted to further validate the assay.

## 5 Conclusion

The KHV-POCT assay demonstrated a total detection time of 65 min, comprising 5 min for nucleic acid release and 60 min for the visualized LAMP reaction. Our results indicate that the KHV-POCT assay possesses sensitivity and specificity comparable to those of the TaqMan qPCR assay. These performance characteristics were corroborated through parallel testing of clinical specimens using both methodologies, which yielded 100% concordant results. Notably, the direct use of nucleic acids released from gill or anal swab samples as templates for LAMP amplification, coupled with a colorimetric readout, facilitated the practical implementation of the KHV-POCT assay. This advancement shows significant potential to enhance the control and management of KHV infections in various aquaculture scenarios.

## Data Availability

The raw data supporting the conclusions of this article will be made available by the authors, without undue reservation.
