# Supplementary material for: Rapid, non-invasive, visual point-of-care detection of koi herpesvirus using loop-mediated isothermal amplification
Source: Front Immunol. 2026 Jul 2;17:1852824. doi: 10.3389/fimmu.2026.1852824 (PMC13372614; doi:10.3389/fimmu.2026.1852824)
Supplement: Supplementary file 1 [file DataSheet1.docx]

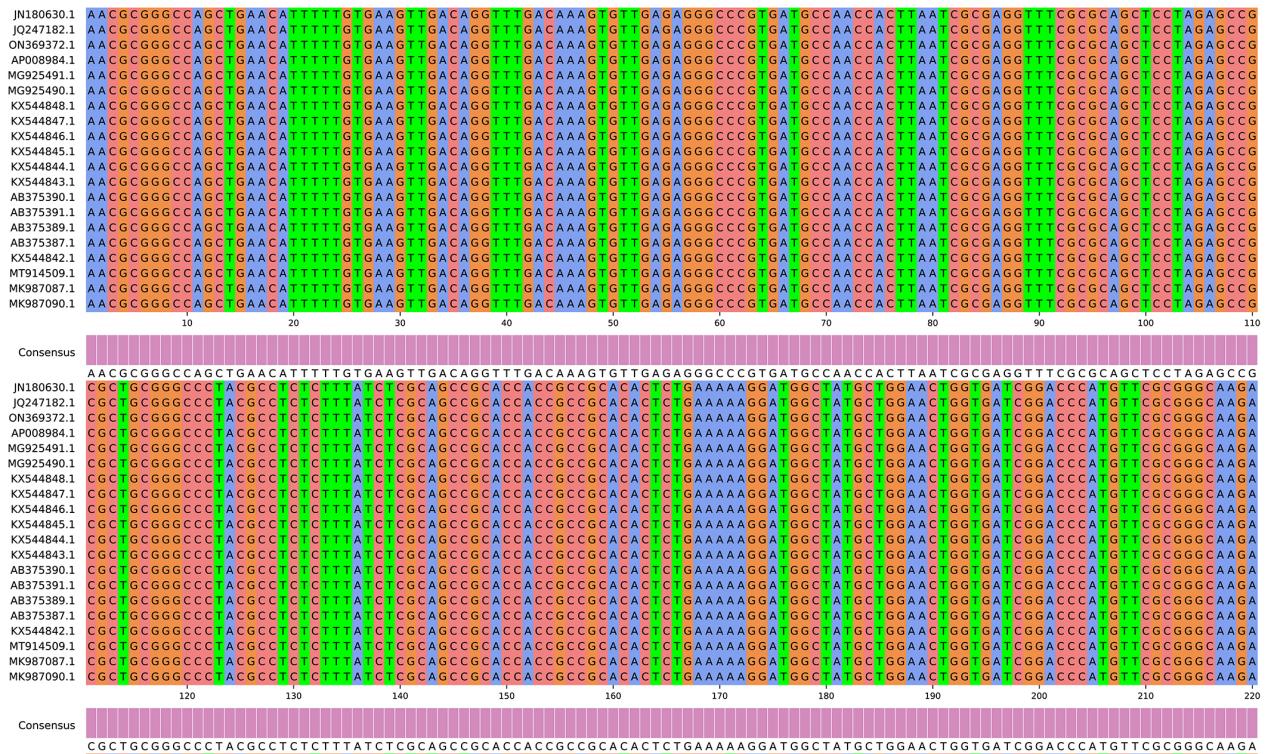


Figure S1. Nucleotide sequence alignment of the TK gene from 20 KHV reference strains.

Table S1. Visual detection of serially diluted positive plasmid.

| **Concentration of positive plasmid**  **(copies/μL)** | **Visual detection result** | | | |
| --- | --- | --- | --- | --- |
|  | **Replicate 1** | **Replicate 2** | **Replicate 3** | **Result** |
| 2 × 10^6^ | red | red | red | + |
| 2 × 10^5^ | red | red | red | + |
| 2 × 10^4^ | red | red | red | + |
| 2 × 10^3^ | red | red | red | + |
| 2 × 10^2^ | red | red | red | + |
| 2 × 10^1^ | red | red | red | + |
| 2 × 10^0^ | yellow | yellow | yellow | - |
| dd H_2_0 | yellow | yellow | yellow | - |

Table S2. Amplification Cp value of nuleic acids from different fish pathogens.

| **Name of fish pathogens** | **Visual detection result** | | | |
| --- | --- | --- | --- | --- |
|  | **Replicate 1** | **Replicate 2** | **Replicate 3** | **Result** |
| DNA of KHV-positive tissue | red | red | red | + |
| KHV-positive plasmid | red | red | red | + |
| CCV | yellow | yellow | yellow | - |
| CEV | yellow | yellow | yellow | - |
| CyHV-2 | yellow | yellow | yellow | - |
| STIV | yellow | yellow | yellow | - |
| RSIV | yellow | yellow | yellow | - |
| WSSV | yellow | yellow | yellow | - |
| *A.veronii* | yellow | yellow | yellow | - |
| dd H_2_0 | yellow | yellow | yellow | - |

Table S3. Evaluation of DNA release effect in mucus specimens via four different methods.

| **Specimen name** | **DNA extration method** | **TaqMan qPCR** | | **LAMP** | | **Visualized LAMP** | |
| --- | --- | --- | --- | --- | --- | --- | --- |
|  |  | **Ct** | **Result** | **Cp** | **Result** | **Color** | **Result** |
| 1G ^!^ | 1 ^a^ | 24.33 | + | 13.79 | + | red | + |
|  | 2 ^b^ | 19.37 | + | 11.49 | + | red | + |
|  | 3 ^c^ | 24.14 | + | 14.69 | + | red | + |
|  | 4 ^d^ | 22.89 | + | 13.29 | + | red | + |
| 1A ^#^ | 1 | 33.23 | + | 16.85 | + | red | + |
|  | 2 | 28.43 | + | 16.62 | + | red | + |
|  | 3 | 30.93 | + | 15.49 | + | red | + |
|  | 4 | 24 | + | 12.6 | + | red | + |
| 2G | 1 | 35.06 | + | 23.63 | + | red | + |
|  | 2 | 30.74 | + | 14.66 | + | red | + |
|  | 3 | 26.07 | + | 13.82 | + | red | + |
|  | 4 | 33.52 | + | 16.21 | + | red | + |
| 2A | 1 | 30.94 | + | 15 | + | red | + |
|  | 2 | 32.95 | + | 16.03 | + | red | + |
|  | 3 | nd * | - | nd | - | yellow | - |
|  | 4 | 31.88 | + | 17.92 | + | red | + |
| KHV-positive plasmid |  | 29.86 | + | 13.6 | + | red | + |
| dd H_2_O |  | nd | - | nd | - | yellow | - |

^!^ G represents gill swab specimen. ^#^ A represents anal swab specimen. * nd means not detected. ^a^ DNA extraction method 1 means the mucus specimens were treated with Release solution 1. ^b^ DNA extraction method 2 means the mucus specimens were treated with Release solution 2. ^c^ DNA extraction method 3 means the mucus specimens were treated with Release solution 3. ^d^ DNA extraction method 4 means the mucus specimens were treated with conventional DNA extraction kit.

Table S4. Detection of clinical specimens via visualized LAMP and qPCR assays.

| **Specimen name** | **Specimen type** | **Visualized LAMP** | | **TaqMan qPCR** | |
| --- | --- | --- | --- | --- | --- |
|  |  | **Color** | **Result** | **Ct** | **Result** |
| 1 | gill | red | + | 25.77 | + |
| 2 | gill | red | + | 19.98 | + |
| 3 | gill | red | + | 22.33 | + |
| 4 | gill | red | + | 26.46 | + |
| 5 | gill | red | + | 24.79 | + |
| 6 | gill | red | + | 22.58 | + |
| 7 | gill | red | + | 20.55 | + |
| 8 | gill | red | + | 24.27 | + |
| 9 | gill | red | + | 17.78 | + |
| 10 | gill | red | + | 19.53 | + |
| 11 | intestine | yellow | - | nd * | - |
| 12 | intestine | yellow | - | nd | - |
| 13 | intestine | yellow | - | nd | - |
| 14 | intestine | yellow | - | nd | - |
| 15 | intestine | red | + | 24.58 | + |
| 16 | intestine | red | + | 30.95 | + |
| 17 | intestine | red | + | 27.96 | + |
| 18 | intestine | red | + | 22.01 | + |
| 19 | intestine | red | + | 23.90 | + |
| 20 | intestine | red | + | 23.81 | + |
| 21 | gill mucus | red | + | 30.26 | + |
| 22 | gill mucus | red | + | 34.09 | + |
| 23 | gill mucus | red | + | 33.36 | + |
| 24 | gill mucus | red | + | 23.48 | + |
| 25 | anal mucus | red | + | 30.77 | + |
| 26 | anal mucus | red | + | 33.38 | + |
| 27 | anal mucus | red | + | 26.05 | + |
| 28 | anal mucus | red | + | 22.12 | + |
| Positive control | / | red | + | 25.72 | + |
| Negative control | / | yellow | - | nd | - |

* nd means not detected.

Proposed POCT Protocol for Non-Invasive KHV Detection in koi

This protocol integrates a one-step sample release procedure with a visualized LAMP assay. Briefly, gill or anal swabs are treated with either of the two release solutions for 5 min at room temperature. Subsequently, 1 μL of the supernatant is aspirated and transferred into the amplification system. The visualized KHV-LAMP reaction is performed in a total volume of 20 μL, consisting of 17 μL of MyLab® visualized LAMP DNA detection solution, 1 μL of Bst DNA polymerase (8 Units), 0.16 μM of primers F3/B3, 1.6 μM of primers FIP/BIP, 0.4 μM of primers F-loop/B-loop, and 1 μL of template. Following incubation at 65 °C for 60 min, the assay results are interpreted directly through visual inspection of color changes: a red color indicates a positive result, whereas a yellow color denotes a negative result.
